# Appropriateness of Antibiotic Prescribing and Its Influencing Factors in Common Clinical Scenarios: A Vignette-Based Cross-Sectional Study From Karaikal, India

**DOI:** 10.7759/cureus.104789

**Published:** 2026-03-06

**Authors:** Kaandeeban Mohanraj, Vidhya Mohanraj, Jinali S Shah, Mohanraj Kalyanasundaram

**Affiliations:** 1 Medicine, Indira Gandhi Medical College and Research Institute, Puducherry, IND; 2 Medicine, United Lincolnshire Teaching Hospitals NHS Trust, Lincoln County Hospital, Lincoln, GBR; 3 Medicine, Narendra Modi Medical College, Ahmedabad, IND; 4 General Practice, Department of Health and Family Welfare, Karaikal, IND

**Keywords:** anti-bacterial agents, antimicrobial stewardship, drug prescriptions, public health policy, surveys, validated questionnaires

## Abstract

Background: Antimicrobial resistance (AMR) is a major global health threat driven by inappropriate antibiotic use. There is a lack of region-specific prescribing data from India. The primary objective of this study was to assess the appropriateness of antibiotic prescribing using standardized clinical vignettes. The secondary objectives were to evaluate factors influencing prescribing decisions and practitioners’ attitudes toward antimicrobial stewardship among medical practitioners in Karaikal, Puducherry, India.

Methods: A cross-sectional study was conducted among 192 practitioners using eight validated clinical vignettes (1,536 prescribing decisions). The appropriateness of prescribing was assessed using national and international guidelines. The influencing factors and stewardship attitudes were evaluated using a five-point Likert scale. Statistical analyses included chi-square tests for categorical variables and Pearson correlation and multivariable linear regression for the total vignette score.

Results: The overall prescribing appropriateness across the eight clinical vignettes was 57.2% (879 of 1,536). Overprescribing was most frequent for physiological leukorrhea, herpes zoster, asthma exacerbation, and upper respiratory tract infection (URTI). Higher appropriateness was observed among superspecialists, government/dual-sector doctors, and those with greater than 10 years of experience (p < 0.001). Experience greater than 10 years has independently predicted appropriateness. Strong support for antibiotic stewardship was also observed.

Conclusion: Prescribing appropriateness improved with seniority, pointing out the need for targeted Continuing Medical Education (CME), guideline dissemination, and stewardship interventions to address AMR.

## Introduction

Antimicrobial resistance (AMR) is a major global public health threat driven largely by inappropriate and unjustified antibiotic use. The burden of AMR is particularly high in low- and middle-income countries, especially in South Asia, with India accounting for one of the highest AMR burdens worldwide [1]. It is estimated that one-third of prescriptions in hospitals are inappropriate. Various systemic and behavioral factors drive antibiotic prescriptions, as evidenced by studies conducted in India [1-3]. The global goal is to reduce the consumption of antibiotics, which is especially concerning in a country like India due to the high infectious burden; inadequacy in the prevention and control of infections; limitations with surveillance; laboratory resources to test susceptibilities; and overburdened healthcare providers [1,2,4-7].

AMR is projected to cause 10 million deaths annually by 2050 if not addressed. The WHO implemented the AWaRe strategy for antibiotic stewardship, which is “Access, Watch, and Reserve," since 2017. Access drugs are easily accessible; watch is carefully prescribed to preserve their effectiveness; and reserve are the last resort choices, with a recent update about drugs that should not be used added to the classification in 2023 to mitigate the burden of AMR [1,8,9]. To avert the emergence of antimicrobial resistance (AMR) and mitigate the consequences of resistance, it is imperative to undertake research, devise interventions, and execute them, particularly in lower-to-middle-income nations like India.

The present study from Karaikal, a mixed public-private healthcare setting within the Union Territory of Puducherry, aimed to evaluate antibiotic prescribing decision-making systematically using standardized clinical vignettes. The primary objective was to assess the appropriateness of antibiotic prescribing in common clinical scenarios. The secondary objectives were to examine factors influencing prescribing decisions and to evaluate medical practitioners’ attitudes toward antimicrobial stewardship practices. These findings are intended to inform targeted antimicrobial stewardship interventions at the regional level.

## Materials and methods

Study design and setting

This cross-sectional, questionnaire-based study assessed the antibiotic-prescribing practices and factors influencing the prescribing decisions of medical practitioners, contributing to efforts to address the growing challenge of antimicrobial resistance. The study was conducted in the Karaikal region of the Union Territory of Puducherry, India, and included registered medical practitioners working in government, private, and dual-sector (government and private) healthcare settings. Data were collected between May 2024 and May 2025. Ethical approval was obtained from the Institutional Ethics Committee of Vinayaka Mission's Research Foundation (approval no. IEC/VMMCH/2024/APR/14).

Study population and sampling 

Registered medical practitioners actively involved in outpatient and/or inpatient care were eligible for inclusion. Practitioners not currently involved in clinical practice, interns, and those who did not provide informed consent or submit incomplete questionnaires were excluded. Participants were recruited using convenience sampling methods. Eligible practitioners were approached in person and invited to participate in the study. A total of 192 medical practitioners participated in this study. The sample size was determined based on feasibility and participant availability and was considered adequate for the exploratory cross-sectional analysis.

Study tool and data collection procedure

Data were collected using a structured questionnaire titled “Pattern of Antibiotic Prescriptions: A Study Among Medical Practitioners." The questionnaire was developed following a review of the relevant literature and was reviewed by subject-matter specialists prior to data collection [10-14]. The questionnaire comprised four sections: demographic information, eight clinical vignettes assessing antibiotic prescription decisions, factors influencing antibiotic prescriptions, and opinions on antimicrobial stewardship practices. The eight validated clinical vignettes generated 1,536 prescribing decisions (192 participants × 8 vignettes).

The validity of the questionnaire was established through expert review by subject-matter specialists affiliated with the medical college in the region, who evaluated the clarity, clinical relevance, and guideline concordance of the clinical vignettes and questionnaire items. Necessary modifications were incorporated based on expert feedback prior to data collection. Table 1 summarizes the details of the clinical vignettes, including the diagnosis and guideline-based appropriateness of antibiotic use.

**Table 1:** Summary of clinical vignettes presented to participants, indicating the final diagnosis, antibiotic indication, and corresponding national or international guideline references used for appropriateness assessment. NCDC, National Centre for Disease Control; ICMR, Indian Council of Medical Research; IAP, Indian Academy of Pediatrics; CHEST, American College of Chest Physicians; NACO, National AIDS Control Organization; URTI, upper respiratory tract infection; ACE, angiotensin-converting enzyme.

Vignette No.	Clinical Scenario (Brief)	Diagnosis	Antibiotics Indicated	Guideline Reference
1	Fever, thrombocytopenia, eschar	Scrub typhus	Yes	NCDC, ICMR
2	Acute cough, coryza	URTI (viral)	No	NCDC
3	Persistent cough on ACE inhibitor	Drug-induced cough	No	CHEST
4	Painful dermatomal rash	Herpes zoster	No	NCDC
5	Wheeze, no infection	Asthma exacerbation	No	NCDC
6	Ear pain with fever	Acute otitis media	Yes	IAP
7	Erythematous swollen limb	Cellulitis	Yes	NCDC
8	White vaginal discharge, asymptomatic	Physiological leukorrhea	No	NACO

Responses, apart from demographics, were measured using five-point Likert scales. Participants were recruited using a convenience sampling approach. The investigators approached registered medical practitioners actively involved in clinical care across government, private, and dual-sector healthcare settings in the Karaikal region in person and invited them to participate voluntarily.

Data were collected daily by the principal investigator and co-investigator. To maximize data collection and minimize non-response, the questionnaire was administered using both paper-based forms and an electronic platform (Google Forms, Google LLC, Mountain View, California, USA). Responses from both modes were combined for analysis.

To minimize bias, participation was voluntary, responses were anonymized, and no identifying information was collected from participants. Standardized clinical vignettes were used to reduce variability in clinical interpretation. The complete questionnaire has been provided in Appendix 1 to enable reproducibility and facilitate use in future studies conducted in similar or different healthcare settings.

Variables studied

The primary outcome was the appropriateness of antibiotic prescribing, assessed using responses to eight clinical vignettes based on national and international treatment guidelines. Secondary outcomes included factors influencing prescribing decisions and attitudes toward antimicrobial stewardship. The independent variables included demographic and professional characteristics such as educational qualification (undergraduate, postgraduate, or superspecialist), gender, years of clinical experience, and practice setting (government, private, or dual-sector). Categorical variables were summarized using frequency and percentage.

Likert-scale responses were treated as approximately continuous variables after summation or averaging. Prescribing decisions based on clinical vignettes were classified as appropriate or inappropriate based on the guidelines. In vignettes in which antibiotics were indicated, strongly agree responses were considered appropriate, whereas neutral, disagree, and strongly disagree responses were considered inappropriate. Reverse classification was applied to vignettes in which antibiotic prescriptions were not indicated. Factors influencing antibiotic prescriptions and attitudes toward antimicrobial stewardship were also assessed. Potential confounders, including clinical experience and practice settings, were considered in the multivariable analyses.

Statistical analyses

Data were entered into Microsoft Excel (Microsoft Corporation, Redmond, Washington, USA) and analyzed using jamovi software (version 2.7.15, jamovi Project, Sydney, Australia) and RStudio (Posit version 2026.01.1+403, Posit Software, PBC (formerly RStudio), Boston, Massachusetts, USA). Categorical variables were summarized as frequencies and percentages, and continuous variables were expressed as mean ± standard deviation, as appropriate. The appropriateness of prescription decisions across practitioner characteristics, including education level, years of experience, and practice setting, was compared using the chi-square test.

For correlation and regression analyses, a total vignette appropriateness score (ranging from 0 to 8) was calculated. A score of 1 was assigned for each prescribing decision that followed the guidelines, whereas incorrect decisions received a score of 0. This method was performed across eight clinical vignettes (Appendix 2).

The association between the appropriateness of prescriptions and influencing factors was evaluated using Pearson’s correlation coefficient. A multivariable linear regression model was constructed to identify independent predictors of prescribing appropriateness, adjusting for demographic variables, clinical experience, practice setting, and factors influencing appropriate antibiotic prescribing. Model assumptions, including normality, absence of multicollinearity, and independence of residuals, were assessed and deemed acceptable. Statistical significance was set at p < 0.05.

## Results

Participant characteristics

A total of 192 medical practitioners participated in this study. Of these, 53.6% (n = 103) were undergraduate MBBS practitioners, 44.3% (n = 85) were postgraduate MD/MS doctors, and 2.1% (n = 4) were superspecialists. The majority of specialists were from internal medicine and pediatrics, with 33 and 19 participants, respectively. Male practitioners constituted 56.8% (n = 109) of the sample.

The mean duration of clinical experience for the doctors was 6.75 ± 8.95 years, and when stratified, 50% had experience of less than three years, followed by three to 10 years and greater than 10 years. Regarding the practice setting, 40.6% (n = 78) worked in the government sector, 40.1% (n = 77) in the private sector, and 19.3% (n = 37) in both sectors. Table 2 summarizes the demographics of the 192 medical practitioners who participated in this study.

**Table 2:** Baseline demographic and professional characteristics of the study participants (N = 192).

Characteristic	Category	n (%)
Educational level	MBBS (Undergraduate)	103 (53.6)
	MD/MS (Postgraduate)	85 (44.3)
	Superspecialty and above	4 (2.1)
Gender	Male	109 (56.8)
	Female	83 (43.2)
Professional experience (years)	<3 years	96 (50.0)
	3-10 years	56 (29.2)
	>10 years	40 (20.8)
Experience of doctors	Mean ± SD (years)	6.75 ± 8.95
Practice setting	Government sector	78 (40.6)
	Private sector	77 (40.1)
	Both government and private	37 (19.3)

Overall appropriateness of antibiotic prescription

**Figure 1:**
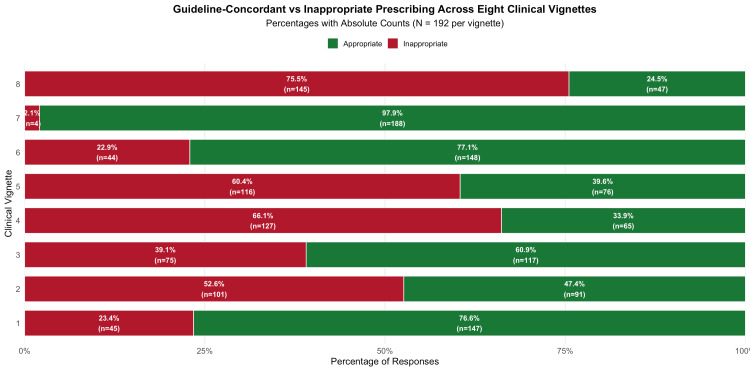
Stacked horizontal bar chart showing distribution of guideline appropriate vs inappropriate responses across eight clinical vignettes. Vignette diagnoses were as follows: scrub typhus (1), viral upper respiratory tract infection (2), ACE inhibitor-induced cough (3), herpes zoster (4), acute exacerbation of bronchial asthma (5), acute otitis media (6), cellulitis (7), and physiological leukorrhea (8). ACE, angiotensin-converting enzyme.

We analyzed 1,536 prescription decisions across eight clinical cases. Overall, 57.2% (879 of 1,536) of decisions were appropriate (Figure 1). In situations where antibiotics were needed, 83.8% (483 out of 576) were prescribed correctly. However, when antibiotics were not needed, appropriate withholding occurred in 41.2% (396 out of 960) of the decisions.

Vignette-specific prescribing patterns

**Figure 2:**
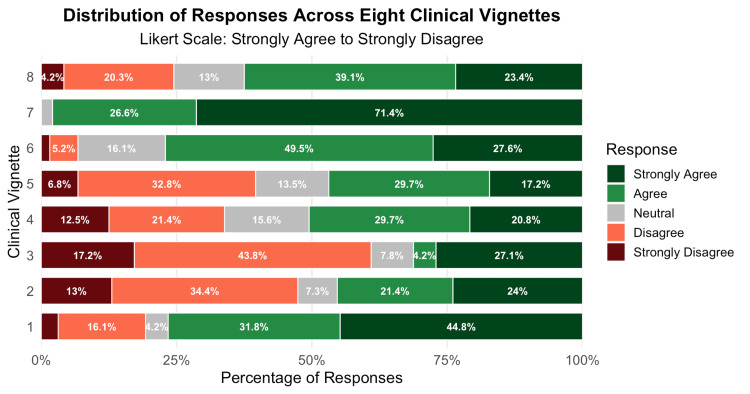
Stacked horizontal bar chart showing the percentage distribution of responses to eight clinical vignettes evaluating antibiotic prescribing decisions based on guideline recommendations. Responses are presented on a five-point Likert scale ranging from strongly agree to strongly disagree. Each bar represents one clinical vignette, with segments indicating the proportion of participants selecting each response category. Vignette diagnoses were as follows: scrub typhus (1), viral upper respiratory tract infection (2), ACE inhibitor-induced cough (3), herpes zoster (4), acute exacerbation of bronchial asthma (5), acute otitis media (6), cellulitis (7), and physiological leukorrhea (8). ACE, angiotensin-converting enzyme.

The appropriateness varied considerably across different clinical vignettes. There were high rates of appropriate prescribing for cellulitis (97.9%, n = 188), acute otitis media (77.1%, n = 148), and scrub typhus (76.6%, n = 147). In contrast, inappropriate antibiotic use was most common in physiological leukorrhea (75.5%, n = 145), herpes zoster (66.1%, n = 127), acute exacerbation of bronchial asthma (60.4%, n = 116), and upper respiratory tract infections (52.6%, n = 101). Figure 2 shows the distribution of responses across the eight clinical scenarios.

Prescribing appropriateness by practitioner characteristics

Among the educational groups, appropriate prescribing was observed in 481 of 824 decisions (58.4%) among MBBS practitioners, 375 of 680 decisions (54.9%) among MD/MS practitioners, and 23 of 32 decisions (71.9%) among superspecialists. With respect to clinical experience, appropriate prescribing occurred in 403 of 776 decisions (51.9%) among practitioners with less than three years of experience, 256 of 440 decisions (58.2%) among those with three to 10 years of experience, and 220 of 320 decisions (68.8%) among practitioners with more than 10 years of experience.

Appropriateness also varied by practice setting. Practitioners working exclusively in the private sector demonstrated appropriate prescribing in 309 of 616 decisions (50.2%), compared to 382 of 624 decisions (61.4%) in the public sector. Those engaged in both public and private practice showed appropriate prescribing in 188 of 296 decisions (63.5%).

Factors influencing prescribing decisions and stewardship attitudes

Laboratory test results (mean = 4.30 ± 0.80), symptom severity (mean = 3.89 ± 0.97), and anticipation of positive outcomes (mean = 3.25 ± 1.06) were the key factors influencing antibiotic prescription decisions. The participants showed strong support for antimicrobial stewardship programs. The strategies of reinforcing standard guidelines; community education regarding antibiotics, antibiotic stewardship, and Continuing Medical Education (CME)/Continuing Professional Development (CPD) programs in healthcare institutions; prevention of over-the-counter sale of antibiotics; and regulation of antibiotic promotion by pharmaceutical representatives all received mean scores above 4.0 on a five-point scale.

Factors associated with prescribing appropriateness

The appropriateness of prescriptions varied significantly based on practice settings and years of clinical experience. Practitioners in the government sector and those in both the government and private (dual sector) groups showed higher levels of appropriateness than those who only worked in the private sector (χ² = 21.4, p < 0.001). Moreover, practitioners possessing over 10 years of experience indicated markedly greater prescribing appropriateness compared to their less experienced counterparts (χ² = 26.4, p < 0.001). No statistically significant difference in overall prescribing appropriateness was observed among the medical practitioners categorized based on their educational qualifications (χ² = 4.45, p = 0.108).

Correlation and regression analyses

When using the Pearson correlation coefficient, a significant negative correlation between the desire to ensure positive patient outcomes and prescribing appropriateness was observed (r = -0.162, p = 0.024). In the multivariate linear regression model, the total vignette appropriateness score was chosen as the dependent variable. Education level, years of clinical experience, practice setting, and factors influencing antibiotic prescription decisions were entered as independent variables. The overall model was statistically significant (R² = 0.217, p < 0.001), explaining 21.7% of the variance in the total appropriateness scores.

**Figure 3:**
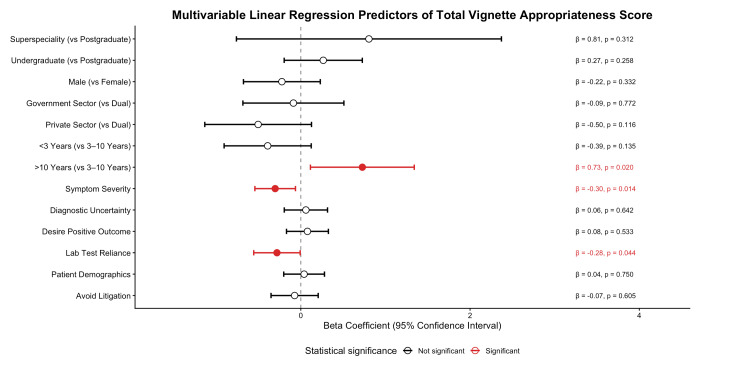
Multivariable linear regression analysis identifying independent predictors of total vignette appropriateness score. Forest plot showing beta coefficients with 95% confidence intervals from multivariable linear regression analysis of predictors of total vignette appropriateness score. Red markers indicate statistically significant predictors (p < 0.05).

As shown in Figure 3, regression analysis showed that clinical experience greater than 10 years was independently associated with higher total vignette appropriateness scores compared to those with less than three years of experience (β = 0.727, p = 0.020). In contrast, greater reliance on laboratory test results (β = -0.282, p = 0.044) and symptom severity (β = -0.303, p = 0.014) were independently associated with lower total vignette appropriateness scores. Educational qualifications and practice settings were not independently associated with appropriateness scores in the regression analysis.

## Discussion

This study demonstrated an overall prescribing appropriateness level of 57.2% across 1,536 prescriptions made by 192 medical practitioners in the Karaikal region of India. Higher appropriateness was observed among practitioners working in public and dual-sector settings and among those with greater than 10 years of clinical experience compared with other subgroups. Notably, no significant differences were observed in the total vignette appropriateness scores across educational categories. Inappropriate antibiotic use was particularly evident in several non-bacterial conditions, including acute upper respiratory tract infections, non-infective exacerbations of bronchial asthma, herpes zoster, and physiological leukorrhea.

Puducherry is a union territory of India consisting of four districts: Puducherry, Karaikal, Mahe, and Yanam, with Karaikal being the second-largest district after Puducherry. According to the Puducherry Year-at-a-Glance statistics, the Union Territory has approximately 3,547 registered medical practitioners, of whom 506 practice in the Karaikal region across all sectors. The present study captured data from nearly 38% of the total practitioner population in Karaikal, supporting the regional representativeness of the findings [15].

Studies deploying different methodologies were used to assess prescription practices in the region of Puducherry, with the majority being pharmacy-based audits. The present study adds to the growing clinical evidence indicating deviations from appropriate antibiotic use in the Union Territory of Puducherry. A recent pharmacy-based prescription audit demonstrated that antibiotic prescription rates exceeded WHO benchmarks, extensive polypharmacy, and predominant use of WATCH category antibiotics among private practitioners [16].

Outpatient pharmacy-based audits conducted in government tertiary care hospitals reported the presence of polypharmacy. Despite partial adherence to WHO antibiotic prescribing standards, antibiotics were prescribed in fewer than 30% of patient encounters in both audits [17,18]. Similarly, another study monitoring antibiotic utilization in public healthcare facilities revealed gaps in rational antibiotic practices despite structured public health settings. The most inappropriate antibiotics are prescribed for viral upper respiratory tract infections [19].

This conclusion is in concordance with our study findings that doctors working in the government or dual sector have better appropriateness of antibiotic prescribing compared to private practitioners. In addition to prescriber- and system-level factors, community-related influences may contribute to irrational antibiotic use in the region. A community-based survey from urban Pondicherry reported inadequate awareness and misconceptions regarding antibiotic consumption, which may indirectly increase patient expectations and the pressure on clinicians to prescribe antibiotics [20].

The findings of the present study are consistent with several questionnaire- and Likert scale-based studies from different regions of India that have examined knowledge, attitudes, and decision-making regarding antibiotic prescription among medical practitioners. Using structured Likert-scale instruments, studies from South India and other regions have similarly demonstrated a discordance between awareness of antimicrobial resistance and actual prescribing behavior, particularly in self-limiting and non-bacterial clinical scenarios. Although a high proportion of physicians in these studies acknowledged antibiotic overuse and supported antimicrobial stewardship interventions, inappropriate justification of antibiotics for conditions such as viral respiratory infections and uncomplicated clinical presentations remains common. These patterns closely mirror those of the present study, where fewer than half of vignette-based prescribing decisions were guideline-concordant, despite strong endorsement of stewardship strategies [21-24].

Studies using Likert-scale assessments consistently associated greater clinical experience and structured practice environments with rational prescribing. Similar to our findings, a study found that physicians with longer practice durations were less likely to endorse antibiotic use in inappropriate scenarios in South Indian teaching hospitals and tertiary care settings [21].

Additionally, diagnostic uncertainty, perceived severity of illness, and concern for patient outcomes emerged as key drivers of antibiotic prescription decisions across studies, aligning with the negative association observed between symptom severity and appropriate prescribing in our regression analysis. Collectively, these studies, including the present investigation, highlight that although attitudes supporting rational antibiotic use are widespread among Indian practitioners, the translation of this awareness into consistent guideline-based prescribing remains incomplete.

The findings of the present study are further supported by converging evidence from a recent multi-country questionnaire-based survey conducted in low- and middle-income countries and a comprehensive systematic review examining factors influencing antimicrobial prescribing decisions. A multi-country survey demonstrated that, despite high levels of awareness regarding antimicrobial resistance and strong endorsement of training and stewardship initiatives, antibiotic prescribing in common clinical scenarios was frequently influenced by perceived illness severity, patient expectations, limited diagnostic support, and concerns regarding adverse outcomes [25].

Complementing this, a systematic review synthesized qualitative evidence identifying diagnostic uncertainty, fear of undertreatment, patient pressure, and reliance on clinical experience as dominant drivers of antibiotic prescribing, particularly in low- and middle-income countries (LMIC) and private-sector settings [26]. These themes closely align with our findings, where perceived symptom severity and reliance on investigations were independently associated with lower prescribing appropriateness, and greater clinical experience predicted more guidelines-concordant decisions. Taken together, these studies reinforce that inappropriate antibiotic use in LMIC contexts is driven less by a lack of awareness and more by contextual, behavioral, and system-level pressures, indicating the importance of antimicrobial stewardship strategies that extend beyond knowledge dissemination to address decision-making environments and prescription incentives.

We should consider the limitations of this study. First, the use of a cross-sectional design and convenience sampling limits causal inference and may restrict the generalizability of the findings beyond the Karaikal region of India. Second, although vignette-based questionnaires are valuable for assessing clinical reasoning and guideline awareness, they may not fully capture real-world prescribing behaviors, particularly in complex or resource-limited clinical settings. Third, social desirability bias may have influenced the self-reported responses, potentially overestimating the appropriateness of prescribing. Additionally, the study did not include prescription audits, microbiological data, or patient outcomes; therefore, it could not directly quantify actual antibiotic exposure or resistance patterns.

Despite these limitations, the use of standardized and validated clinical vignettes, inclusion of practitioners from diverse practice settings, and integration of attitudinal and behavioral factors strengthened the study’s internal validity. Future research should adopt mixed-methods approaches, integrating vignette-based assessments with prescription audits, microbiological surveillance, and qualitative interviews to more effectively link prescribing attitudes with real-world clinical practices. Furthermore, implementing routine prescription audits alongside targeted community-level awareness initiatives on antibiotic use may serve as an effective strategy to address this issue at both the healthcare system and community levels. These findings also contribute to the broader global effort to optimize antibiotic use in low- and middle-income countries facing a growing antimicrobial resistance burden.

## Conclusions

Overall, guideline-concordant antibiotic prescribing among medical practitioners in Karaikal was modest, with 57.2% (879 of 1,536) of decisions deemed appropriate. Although antibiotics were appropriately prescribed when indicated, appropriate withholding occurred in only 41.2% (396 of 960) of decisions in non-bacterial scenarios, highlighting overprescribing as the primary concern. Inappropriate use was most evident for viral upper respiratory infections, asthma exacerbations, herpes zoster, and physiological leukorrhea. More than 10 years of clinical experience independently predicted higher appropriateness.

These findings underscore the need for targeted antimicrobial stewardship strategies that address the behavioral drivers of prescribing, particularly among early-career and private-sector practitioners. The supportive responses from practitioners regarding possible strategies to tackle this growing problem represent their positive attitude. Collectively, such regional efforts may contribute to regional and national progress toward achieving global antimicrobial resistance (AMR) targets.

## Appendices

Appendix 1

Study Questionnaire

Part A: Demographics

1. Education: ☐ Undergraduate (MBBS) ☐ Postgraduate (MD/MS) ☐ Super specialty and above

2. If postgraduate and above, please specify specialty: ___________________________

3. Gender: ☐ Male ☐ Female ☐ Other

4. Years of Clinical Experience: ___________________________

5. Working Setup: ☐ Public sector ☐ Private sector ☐ Both

Part B: Clinical vignettes

For each of the following scenarios, indicate your level of agreement with the statement provided using the five-point Likert scale:

Response options: Strongly Agree / Agree / Neutral / Disagree / Strongly Disagree

1. A 65-year-old male agricultural labourer presented with high-grade fever and myalgia for 10 days. He has a history of sleeping on grass and owning a goat. On examination, he was febrile and a painless eschar was noted in the groin.

I will choose to prescribe an antibiotic for this patient.

☐ Strongly Agree ☐ Agree ☐ Neutral ☐ Disagree ☐ Strongly Disagree 

2. A 32-year-old male presents with dry cough, fever, and body pain since yesterday. Examination findings are normal.

I will choose to prescribe an antibiotic for this patient.

☐ Strongly Agree ☐ Agree ☐ Neutral ☐ Disagree ☐ Strongly Disagree 

3. A 65-year-old hypertensive female presents with a one-week history of intermittent dry cough. Respiratory examination is normal. She has been on amlodipine long-term and ramipril was added two weeks ago.

I will choose to prescribe an antibiotic for this patient.

☐ Strongly Agree ☐ Agree ☐ Neutral ☐ Disagree ☐ Strongly Disagree 

4. A 50-year-old diabetic female presents with severe burning pain over the left loin for three days. Examination reveals multiple vesicles distributed over the left loin up to the midline anteriorly and posteriorly.

I will choose to prescribe an antibiotic for this patient.

☐ Strongly Agree ☐ Agree ☐ Neutral ☐ Disagree ☐ Strongly Disagree 

5. A 45-year-old female presents with acute onset cough and dyspnoea. Cardiovascular examination is normal; bilateral wheeze is present on respiratory examination. She reports similar past episodes.

I will choose to prescribe an antibiotic for this patient.

☐ Strongly Agree ☐ Agree ☐ Neutral ☐ Disagree ☐ Strongly Disagree 

6. A 5-year-old boy presents with sudden onset right earache since evening. He had a sore throat for two days. Examination shows a normal external ear and a bulging tympanic membrane.

I will choose to prescribe an antibiotic for this patient.

☐ Strongly Agree ☐ Agree ☐ Neutral ☐ Disagree ☐ Strongly Disagree 

7. A 27-year-old male presents with swelling of the left foot extending to the ankle, associated with pain and fever following a road traffic accident. Examination shows warmth, tenderness, purulent discharge, and enlarged left inguinal lymph nodes.

I will choose to prescribe an antibiotic for this patient.

☐ Strongly Agree ☐ Agree ☐ Neutral ☐ Disagree ☐ Strongly Disagree 

8. A 27-year-old unmarried female presents with white, foul-smelling vaginal discharge for one week. She is mid-menstrual cycle. Gynecological examination findings are normal.

I will choose to prescribe an antibiotic for this patient.

☐ Strongly Agree ☐ Agree ☐ Neutral ☐ Disagree ☐ Strongly Disagree 

Part C: Deciding factors in antibiotic prescription

Indicate your level of agreement for each factor:

Response options: Strongly Agree / Agree / Neutral / Disagree / Strongly Disagree

1. Severity of symptoms

☐ Strongly Agree ☐ Agree ☐ Neutral ☐ Disagree ☐ Strongly Disagree 

2. Uncertainty about diagnosis

☐ Strongly Agree ☐ Agree ☐ Neutral ☐ Disagree ☐ Strongly Disagree 

3. To ensure a positive outcome

☐ Strongly Agree ☐ Agree ☐ Neutral ☐ Disagree ☐ Strongly Disagree 

4. Laboratory test reports (elevated total WBC and polymorph count)

☐ Strongly Agree ☐ Agree ☐ Neutral ☐ Disagree ☐ Strongly Disagree 

5. Patient demographics (socioeconomic status, education level, access to healthcare, health insurance)

☐ Strongly Agree ☐ Agree ☐ Neutral ☐ Disagree ☐ Strongly Disagree 

6. To avoid litigation

☐ Strongly Agree ☐ Agree ☐ Neutral ☐ Disagree ☐ Strongly Disagree 

Part D: Opinion on safe antimicrobial practices

Indicate your level of agreement:

Response options: Strongly Agree / Agree / Neutral / Disagree / Strongly Disagree

1. CME/CPD programmes on safe antimicrobial practices

☐ Strongly Agree ☐ Agree ☐ Neutral ☐ Disagree ☐ Strongly Disagree 

2. Educating the community on antibiotic use

☐ Strongly Agree ☐ Agree ☐ Neutral ☐ Disagree ☐ Strongly Disagree 

3. Preventing over-the-counter sale of antibiotics

☐ Strongly Agree ☐ Agree ☐ Neutral ☐ Disagree ☐ Strongly Disagree 

4. Publication of standard treatment guidelines

☐ Strongly Agree ☐ Agree ☐ Neutral ☐ Disagree ☐ Strongly Disagree 

5. Implementation of antibiotic stewardship programs in healthcare institutions

☐ Strongly Agree ☐ Agree ☐ Neutral ☐ Disagree ☐ Strongly Disagree 

6. Pharmaceutical companies should include cautionary messages regarding routine antibiotic use during promotional activities

☐ Strongly Agree ☐ Agree ☐ Neutral ☐ Disagree ☐ Strongly Disagree

Appendix 2

**Table 3:** Evaluation and scoring of clinical vignettes.

Component	Description
Guideline Reference	Responses evaluated against national and international treatment guidelines
Vignettes where antibiotics were indicated	Vignettes 1, 6, and 7. “Strongly Agree” and “Agree” were considered guideline-concordant.
Vignettes where antibiotics were not indicated	Vignettes 2, 3, 4, 5, and 8. “Disagree” and “Strongly Disagree” were considered guideline-concordant.
Neutral responses	Categorized as non-concordant for primary outcome analysis.
Overall appropriateness	Calculated as the proportion of guideline-concordant responses among total vignette-based prescribing decisions.
